# Perceptions of patients regarding diabetes-related health communication strategies in the Free State, South Africa

**DOI:** 10.4102/hsag.v24i0.1089

**Published:** 2019-01-08

**Authors:** Champion N. Nyoni, Marianne Reid

**Affiliations:** 1 Paray School of Nursing, Thaba-Tseka, Lesotho; 2 School of Nursing, University of the Free State, South Africa

## Abstract

**Background:**

This study researched the perceptions of patients diagnosed with diabetes concerning diabetes-related health communication strategies in the Free State province in South Africa. The prolongation and quality of life of patients diagnosed with diabetes are affected by lifestyle choices. An enabler of risk reduction is health communication which informs, influences and motivates individuals to adopted health-focused lifestyles.

**Aim:**

This study sought to describe the perceptions of patients regarding diabetes-related health communication strategies in the Free State, South Africa.

**Setting:**

This study was carried out in primary health care centres and community health care centres within the Free State province in South Africa.

**Methods:**

A qualitative, descriptive and exploratory research design was used in this study. Thirty-four patients diagnosed with type two diabetes for at least a year were purposively included in this study. Semi-structured interviews in Afrikaans, English, Sotho and Xhosa were conducted. Data analysis was through inductive reasoning and thematic analysis.

**Results:**

The majority of the respondents were older women having been diagnosed with diabetes for more than 5 years, with at least primary school education and of diverse South African ethnicities. The main prompting questions operationalised the term ‘perception’, probing their feelings, experiences and knowledge of health-related communication strategies as presented by a variety of information sources. After recording interviews, data were analysed according to themes, categories and sub-categories.

**Conclusions:**

The study highlights factors that encourage patients to seek help and foster attitudes of compliance. Practical problems regarding the management of diabetes are underlined. The role of family, as well as the patient–caregiver relationship, in the acceptance and management of the disease is revealed. Societal perception of male symptomology is shown. The study offers information to stakeholders and health care workers for continued successful management of diabetes in communities.

## Introduction

Lifestyle disorders, such as type 2 diabetes mellitus, are on the rise globally. The International Diabetes Federation (IDF) estimates that 629 million people globally will be living with diabetes mellitus by 2045 (IDF 2016). The larger portion of that estimated population will be from low- and middle-income countries, like South Africa. These countries are characterised by limited resources which influence the nature of the strategies they engage in improving the health outcomes of their populations.

Patients with chronic lifestyle disorders, such as diabetes mellitus, are expected to be intensely involved in the management of their condition. The World Health Organization (WHO) underscores the quintessential role people play in determining their own health and ultimately their own future regarding illness and disease (WHO 2016). The IDF ratifies this understanding towards patients’ role in their own health when they state that ‘patients have the right to autonomy, informed decision and to participate in their own care’ (IDF 2016). Therefore, patients need to be empowered with appropriate knowledge and skills to enable them to participate fully in their own self-management.

Strategies used in communicating health information to patients influence their ability to retain and use such health information for positive health outcomes (Vermund et al. 2017). Dominant strategies for communicating health information are usually designed and focused on the health care worker, with limited involvement of the patients who are expected to improve their health outcomes. This is contrary to the promulgations by the WHO (2016) and IDF (2016) that support patient-centred health communication.

Patient-centred health communication strategies are influenced by the self-determination of patients. Self-determination and self-management are based on the premise that people have the capacity to make choices and are responsible for the consequences of their choices (Raaijmakers et al. 2014). The role of the health care worker within such a perspective is transformed from being central in the health of their patients to becoming a more supportive collaborator, who provides opportunities for improved health through communication strategies such as health dialogue (Reid et al. 2018).

Health communication strategies should be embracing patient-centred principles nested within a participatory paradigm aimed at benefiting the patients, improving their capacity and competence in managing their own health, ultimately improving their own health outcomes.

### The context of the study

The South Africa health care system battles with a notorious Quadruple Burden of Disease (QBoD). The QBoD includes the human immunodeficiency virus (HIV)/AIDS epidemic alongside the high burden of tuberculosis; high maternal and child mortality; high levels of violence and injuries; and the burden of non-communicable diseases. In 2016, 57.4% of all deaths in South Africa were attributed to non-communicable diseases, while 5.5% of all deaths were because of diabetes mellitus (Statistics South Africa 2016). Diabetes mellitus is the second deadliest condition in South Africa after tuberculosis.

Primary health care is the vehicle in which health services, such as diabetes-related care, are provided to the population in South Africa. This approach is a tiered interrelated system, linking communities to their health care system. Primary health care clinics (PHCs) are the initial entry of patients into the health delivery system, and such clinics are usually dotted around communities. The PHCs link with the community health centres (CHCs) that represent the next level of care. The focus of the CHCs is on more complex cases that may include maternity services and minor surgeries like suturing. Patients may be transferred to district hospitals and, in some cases, even tertiary hospitals for more advanced and specialised care (Mojaki et al. 2011).

In addition to the disease burden, a serious challenge that the health delivery system is facing is the available workforce within the health delivery system. In as much as South Africa is said to have an adequate health workforce, above the recommended standard set by the WHO (2016), the quality, skill mix and uneven distribution of health care workers between the private and public health systems remain a challenge (Van Rensburg 2014). Compounding such a challenge is the inadequate resources in health centres and health care worker burnout (Klopper et al. 2012). This combination of challenges in the health delivery system in South Africa influences and affects the ability of the entire health delivery system to meet the needs of patients with diabetes through apt health communication strategies.

A health communication strategy encompasses the following: sources of health information, the content of the health information and how the health information is conveyed, including where and when it is communicated (Storey et al. 2014). In the Free State province, diabetes-related information is communicated through a variety of curatively focused health communication strategies. Health care workers, other patients with diabetes and specific people, for example, traditional healers and the clergy, communicate information related to diabetes to patients diagnosed with diabetes. The focus of the communicated information is mainly about nutrition, medication adherence and, in some cases, troubleshooting problems that arise as a result of diabetes. This health information is communicated, on one hand, at patient waiting and consultation rooms enshrined in the concept of health education, while, on the other hand, health information may be communicated in the streets through health campaigns. Electronic and print media in the form of the radio, television and pamphlets are also used to present diabetes-related health information (Krige & Reid 2017). Such information is communicated within a short period of time and may not be tailor-made for specific individuals.

## Problem statement

The health outcomes of patients with diabetes mellitus in South Africa need to be improved through engaging the patients in patient-centred health communication approaches. Persuh (2018) defines perception as the extract of perceptual information that is represented at the personal level and can be used for the control and guidance of individual actions. This may include a recognition and interpretation of sensory and cognitive information gleaned from lived experience and phenomena. Understanding the perceptions of a population, like patients with diabetes in the Free State province reveals the meaning patients make of the health communication strategies. This meaning is essential in the design, development and implementation of a health communication strategy such as a health dialogue model. There is a dearth in literature reflecting perceptions of patients regarding diabetes-related health communication strategies in the Free State. This study forms part of the initial studies in the development of a complex model of health dialogue for patients with diabetes mellitus in the Free State province in South Africa.

## Purpose of the study

This study describes the perceptions of patients regarding diabetes-related health communication strategies in the Free State, South Africa.

## Research method

### Research design

The perceptions of patients regarding diabetes-related health communication strategies in the Free State province were determined through a qualitative, descriptive and exploratory research design.

### Population

The population in this study consisted of all patients diagnosed with type 2 diabetes mellitus accessing health care from all the CHCs (*n* = 10) in the Free State, and PHCs (*n* = 12) in Mangaung sub-district. Mangaung sub-district was included in this study as it has more PHCs when compared with other districts in the Free State, and was logistically accessible for the researchers. The researchers could not accurately establish the number of patients diagnosed with type 2 diabetes mellitus in these sites.

### Unit of analysis

The unit of analysis met the following inclusion criteria, namely that all participants:

- had a medical diagnosis of type 2 diabetes for a minimum of a year as evidenced by their medical record
- had to be above the age of 18 years, which was verified through their legal identity documents
- were attending a PHC or CHC within the Free State province on the date of data collection
- spoke English, Afrikaans, Sesotho or Xhosa.

Participants were included in this study through purposive sampling.

### Data collection

Data were collected through semi-structured interviews from the unit of analysis between 07 and 17 April 2014. The semi-structured interview questions were formulated by operationalising the construct ‘perceptions of diabetes-related health communication strategies’. The semi-structured questions included the following:

- Tell me what you know about diabetes.
- How and by whom was this knowledge related to you?
- Which information was helpful or not helpful to you and why?
- Tell me why the information you received motivated you to change or not to change your health behaviour.

Access to the data collection sites, namely the PHCs and CHCs in the Free State province, was granted by the Free State Provincial Department of Health (PDOH). A schedule for data collection was organised through the professional nurse in charge of the health centre, based on logistical realities of both the researcher and the data collection site. Data in English, Xhosa and Sesotho were collected by the first author who is experienced in conducting interviews, while data in Afrikaans were collected by a research assistant who is an experienced qualitative data researcher. The research assistant was informed of the overall purpose of the study and trained on the data collection process. Each site was identified using the first letter of the district and whether it was either a PHC or a CHC as P or C.

The purposively selected participants were made aware of the purpose of the study, and consent to be part of the study was sought from them (see Table 1). The semi-structured interviews were conducted in a quiet room within the PHCs or CHCs, while a Livescribe^®^ pen was used to record the interviews. Preliminary data analysis was conducted after each interview to enhance the quality of the subsequent interviews and this approach informed data saturation. Data were collected through a stepwise approach, from one data collection site to the next, until data saturation was reached. Data saturation occurred at the ninth CHC and sixth PHC site.

**TABLE 1:** Data collection sites and participants.

District	Number of participants
**District of the Free State (CHCs)**	
FezileDabi	12
Lejweleputswa	3
Motheo	3
Thabo Mofutsanyane	2
**Mangaung sub-district (PHCs)**	
Bloemfontein	4
Botshabelo	5
ThabaNchu	5

**Total**	**34**

CHC, community health centres; PHC, primary health care clinics.

### Data analysis

The recorded semi-structured interviews were transcribed verbatim, and translations to English were done for interviews conducted in the other languages. The transcribed data were then analysed through inductive reasoning integrating frameworks by Creswell (2009), Tesch (1990) and using Atlas.ti^TM^ as a platform for analysis. The data analysis process proceeded in two cycles according to the generic approach to coding (Saldana 2009:48).

The first cycle of data coding integrated six data coding methods, namely open coding, structural coding, initial coding, attribute coding, values coding and *in vivo* coding to generate codes from the interview transcripts. The generated codes were analysed in the second cycle through pattern coding to generate themes and categories which are presented as the results of the study.

### Ethical considerations

Permission to conduct the study was sought from and granted by the Ethics Committee of the Faculty of Health Sciences of the University of the Free State under an overarching study aimed at developing a health dialogue model for patients with diabetes in the Free State, SA (ECUFS NR 39/2013). The Free State Department of Health granted access to all data collection sites.

The study was guided by the three ethical principles underpinning the Belmont report of 1978, namely beneficence, respect for persons and justice (United States Department of Health and Human Services [USDHHS] 1978). Individual consent was sought from the participants after having explained to them the purpose, benefits and limitations of the study. The generated data were stored in a password-protected folder, with only the authors having access to the data.

## Rigour of the study

The study applied the trustworthiness framework in ensuring rigour (Lincoln & Guba n.d.). The constructs underpinning the trustworthiness framework were applied through prolonged engagement with participants until data saturation, space and source triangulation where data were generated from multiple sites within the Free State province. Data analysis followed frameworks of reputable theorists and was applied within ATLAS.ti^TM^ to enhance consistency in data analysis.

## Pilot study

A pilot study was conducted on two patients meeting the inclusion criteria at a PHC clinic in Bloemfontein, Mangaung district. Preliminary analysis of the data from the pilot study revealed clarity and consistencies within interview questions; therefore, no changes were made to the data collection tool. Data from the pilot study were included in the main study.

## Results

Health communication strategies are diverse and their main purpose is to stimulate and inspire patients diagnosed with diabetes in improving their decision-making and self-management related to their health. Two themes emerged from the data and these were supported by various categories and sub-categories. Direct quotations from patient interviews are presented as evidence based on the district where the interview was conducted, whether the interview was conducted in a PHC or CHC and the sequential number of the patients’ interview.

### Theme 1: Guidance towards improved health outcomes

The participants in this study perceived health communication strategies as guidance towards improved health outcomes. Such guidance may be understood as providing direction to a patient with diabetes in need of health information and support for a variety of reasons ultimately leading to improved health outcomes. This theme was supported by five categories, namely motivation to seek health information, the content of health information, sources of diabetes-related health information, techniques used to convey health information and evaluating the effects of health communication strategies.

#### Category 1: Motivation to seek health information

Participants experiencing diabetes-related physical changes and symptoms were prompted to seek knowledge and explanations regarding the changes. One of the participants stated:

‘I went to the toilet an awful lot, lost a lot of weight was very tired and then I told my mom… I thought I had a UTI.’ (MP1, female, 56 years old)

Physical changes that were thought to be harmless were ignored, and those that were perceived as harmful motivated the need to take action. One of the participants stated:

‘I initially ignored the signs … carried on with my life… until they started happening frequently. That got me worried.’ (LC2, male, 47 years old)

Participants would access and evaluate memory, personal experience and gained knowledge regarding how to manage symptoms. When they perceived their knowledge to be inadequate, they were motivated to seek clarification. One of the participants stated:

‘I then realised, if someone does not explain what is happening to me… I really will die.’ (FC1, female, 68 years old)

#### Category 2: The content of health information

The participants in this study stated that they received information focusing mainly on nutrition and how to modify their lifestyle. They knew which diet was expected to be followed including the frequency of their meals. Two participants stated that:

‘They said I shouldn’t eat meat…chicken and I should not eat potatoes… I should not take drinks with gas (fizzy drinks) … I should not eat a lot of papa [*maize meal based thick porridge*].’ (LC2, male, 47 years old)

‘Doctors told me to be particular with what I eat, I shouldn’t skip meals, eat fatty and foods with seasons with salts.’ (TC1, male, 58 years old)

Being diagnosed with diabetes results in lifestyle-related changes (Chong et al. 2017). Participants in this study stated that information provided to them focused on lifestyle modifications associated with diabetes. The modification of lifestyle was focused on certain aspects of self-care. Such information empowered the patients to make decisions that support positive health outcomes. One of the participants highlighted that:

‘And if it happens that you have an injury, it might not be curable. Yes, they have advised us on the type of shoes to wear.’ (FC6, female, 45 years old)

Lifestyle modifications enhance positive health outcomes in patients with diabetes (Group 2017). Participants in this study related that they were aware of specific lifestyle situations that enhance the progression of diabetes. In avoiding progression of their diabetes, the participants claimed to have also learned strategies for avoiding specific situations that might result in ill health or enhance the physiological effects of diabetes. One of the participants relayed some of the content related to such lifestyle modifications:

‘…just like that, they told me that too much stress, depression, push it upwards. So make sure that your level is good… don’t allow people to stress you, just keep your level low.’ (LC4, female, 29 years old)

#### Category 3: The sources of health information

A variety of sources of health information provided guidance related to diabetes in the Free State. The various sources of health information were classified into either direct or indirect sources as influenced by the following criteria, namely the channel of communication used, the nature of interaction and shared objectives between the sources of health information and the patients receiving health information.

The direct sources included a broad spectrum of health care workers, including medical officers, nurses and nutritionists. Family members and patients with or without diabetes shared experiences and health information. The health information sharing happened in the waiting room.

Patients asked their direct sources of diabetes-related questions on issues that seemed unclear and related to specific challenges:

‘Nurses gave me information regarding sugar diabetes… she told me not to eat red apples but [*to eat*] green apples. … we also ask from each other, when we meet at the clinic.’ (LC5, female, 57 years old)

Indirect sources of information were gained from social media:

‘I absolutely go on to find out, I go on Facebook, for example.’ (MP4, female, 28 years old)

#### Category 4: Techniques used to convey health information

Participants related that the voice tone and demeanour of health care workers influenced their reception of the diabetes-related health information. Health care workers that portrayed a more serious and blunt affect were perceived as being effective in transmitting health information. Such health care workers presented health information in a serious manner coupled with appropriate body language for patients to notice.

‘…within five years, they will bury you and he said this with a stern face… I knew he meant business.’ (TC4, male, 40 years old)

Conversely, patients perceived health care workers as being annoyed when patients struggled to understand health information regarding their disease and compliance with prescribed health care behaviour.

‘… here it’s like you annoy them… they quickly want you gone.’ (LC3, female, 38 years old)

The success of diabetes-related health communication was influenced by the language in which it was presented. Mother tongue information was better received and understood:

‘He was very interesting, because he spoke Sesotho, so I related my story like I am doing right now…’ (FC6, female, 45 years old)

Printed media, such as leaflets, text and graphics, are available in the public domain. The radio is often used as a means of communication. These forms of health information are provided in English which is mostly the second or third language of patients. Consequently, they rely on their children to clarify the information:

‘…sometimes when we get there they give us some papers, when I get home, I give it to my children since it will be written in English… I know that they know English, so they will explain to me.’ (LC3, female, 38 years old)

Unfortunately, the problem of male erectile dysfunction (ED) being a symptom of diabetes is also broadcast in the media, especially the radio. This information was received by male patients with diabetes as leading to stigmatisation and rendered the radio as an unfavourable medium to convey health information:

‘Ah, it’s not to build, it is just to criticise, you see? [*they say*] “you cannot erect; you see? You cannot sleep with a woman, you see? … So we’ll come and help you make babies” you see those kind of things.’ (FC4, male, 51 years old)

‘…no, they [*radio*] damage me… because whatever they talk about is related to me. They even mean that I am like this; I live like this…according to what they say.’ (TC2, male, 53 years old)

#### Category 5: Evaluating the effectiveness of health communication strategies

In health counselling, questions by patients are taken as a sign of wanting to reach a better understanding of the problem being discussed and thus accessing information from their health information source. Conversely, health care workers ask questions of patients to establish the level of knowledge, comprehension and retention. Questions serve to generate curiosity from patients regarding the disease. However, cultural practices among patients regard questioning a health care worker as a demeaning practice that negates health care worker’s status. Within this context, asking questions to persons of authority or elders is perceived as demeaning and derogatory:

‘No, I don’t ask them any questions. I don’t ask questions… [*because*] Sesotho is not interpreted’. (MP8, female, 25 years old)

Conversely, health care workers take exception to their advice and instructions being questioned by patients:

‘We tell this person to take their pills and now she is questioning us? You see.’ (TC5, female, 42 years old)

### Theme 2: Adaptation to health information in self-management

Self-management is the ability to care for oneself with minimal aid from external people such as health care workers (O’Donnell et al. 2017). Adaptation to health information in self-management as a theme was supported by categories, namely factors influencing the adaptation to health information in self-management and modifying my lifestyle.

Factors influencing the adaptation of health information in self-management were supported by two sub-categories: interpersonal factors and intrapersonal factors. The category of modifying my lifestyle was divided into two sub-categories: changes in my diet and outcomes of lifestyle modification.

#### Category 1: Factors influencing adaptation of health information in self-management

The ability to conduct self-management was influenced by two main sub-categories: interpersonal and intrapersonal factors.

#### Subcategory 1: Interpersonal factors

Interpersonal factors included all the dynamics of self-management external to the patients diagnosed with diabetes such as family, other patients with diabetes mellitus, religion and access to health care.

Although diabetes is present in a family, other family members may have a genetic predisposition and lifestyle inclination to develop the disease (Pociot & Lernmark 2016). Conversely, family support – emotional, financial and practical such as reading health information for the patient – plays an important role in patients’ coping skills.

In this study, the influence of family members with a similar condition seemed critical in modelling appropriate behaviour and self-management. Thus, the family created a safe environment where the participants were able to discuss self-management related to their conditions:

‘I have children there, they are able to read so they read for me.’ (TC3, female, 61 years, old)

‘…no one ever touches that topic, I only talk with my family members.’ (LC5, female, 63 years old)

Contrary to the notion that family was a safer place for patients with diabetes to discuss their condition, some of the participants said they were selective of issues they discussed with their family. Sensitive concerns were better discussed with other patients with diabetes. One of the male participants who was experiencing ED claimed to feel safe and confident to discuss his challenges with other patients, specifically males diagnosed with diabetes. One of the male participants related his experience with ED:

‘I can’t perform anything because it has eaten my wings, but as we chat with other men they will tell you that it has also eaten their wings, I can’t do anything. I am not the only one though I thought I was.’ (FC4, female, 70 years old)

Other patients experiencing a similar predicament seemed to calm such participants, allowing them to engage in self-management. Participants in this study also mentioned that their religion influenced their ability to adopt health information for their self-management. The nature of the influence was based on the perceived role of God in their treatment options and their will to conduct self-management. Some of the participants reflected that their self-management was purely dependent on their religion with little influence of their own efforts. One participant was quoted as saying:

‘God can heal, not a certain man, God can heal according to his will.’ (LC1, male, 38 years old)

Incorporating health information in self-management of diabetes takes place within the context of participants receiving care in public health facilities. Some of the CHCs and PHCs included in this study seemed to avoid a ‘supermarket’ approach to the provision of diabetes care preferring a specific day within the week to manage patients with similar conditions. Participants in this study perceived these specific days for care within the week to be non-negotiable and missing such days resulted in punitive consequences affecting self-management of patients. One of the participants stated:

‘… but sometimes I miss Thursday, like last week, I had to attend to a friend of mine’s funeral and then I had to wait till today [*to receive care*].’ (TC4, female, 38 years old)

#### Subcategory 2: Intrapersonal factors

Internal driving forces affected the execution of self-management. Positive internal thought processes resulted in perceived positive health outcomes evidenced by the acceptance of being diagnosed with diabetes. These positive internal influences enabled the participants to take necessary measures that enhanced self-management and improved adherence to their treatment schedule. Some of the participants stated:

‘I followed her orders without hesitation because I wanted to be treated.’ (MP7, male, 54 years old)

Contrary to positive thought processes, some thought processes resulted in negative health outcomes. Negative health outcomes included poor adherence to the treatment regimen, poor health outcomes and difficulty in adjusting to the chronic condition.

‘…still today, I can’t accept that I am diabetic. So it’s very hard for me.’ (MP2, female, 32 years old)

‘…I would rather have AIDS than sugar diabetes.’ (MP8, female, 28 years old)

Memory impairment was perceived as influencing the application of health information in self-management of the disease. In this study, some participants tended to forget certain essential health information related to self-care, leading to possible detrimental health outcomes. Some of the participants reflected:

‘I forget too much, it’s a matter of forgetfulness…’ (TC3, female, 61 years old)

#### Category 2: Modifying my lifestyle

The theme self-management was also supported by the category modifying my lifestyle. In this particular study, two sub-categories reflected lifestyle modifications, namely changes in my diet and the outcomes of lifestyle modification.

**Subcategory 1: ‘Changes in my diet’:** The participants in this study modified their diet and noticed a difference in the food they ate before and after being diagnosed with diabetes. This perceived difference allowed participants to reflect on different types of food and their preparation. Challenges associated with dietary modification, such as tastelessness, being expensive and unavailable in local shops, resulted in non-compliance. One of the participants highlighted:

‘…those foods are ugly and tasteless.’ (MP1, female, 56 years old)

Another problem faced by the participants in this study, as they modified their lifestyle in relation to their diet, was the context in which they were to modify their diets. Families faced economic burdens related to dietary modifications and one of the participants expressed this dilemma:

‘You will find that, since we are living together, I’ll be cooking my own dish and you doing the same, you get me… that is expensive.’ (FC4, male, 51 years old)

**Subcategory 2: Outcomes of lifestyle modification:** Several positive outcomes specific to the impact of health communication strategies were reflected by the participants. These participants perceived improved physical health, improved health literacy and positive mental adjustments. Some of the participants highlighted:

‘I know what to use, to what extent, this I don’t use…’ (MP7, female, 56 years old)

‘I just assumed I will stay on the diet that I am on. It works it seems to me.’ (TC6, female, 38 years old)

## Discussion

The purpose of this study was to describe the perceptions of patients regarding diabetes-related health communication strategies in the Free State province of South Africa. The need to understand how patients perceived health communication strategies is essential for the designing and implementation of a patient-centred health communication model nested in a participatory paradigm.

Patients expressed the need to be guided towards improved health outcomes because of a variety of combined factors associated with diabetes mellitus. The initial changes associated with diabetes were not accurately interpreted by patients, thereby motivating them to seek guidance. Failure to accurately interpret physical changes associated with diabetes influences the motivation to seek health care and may be detrimental to the patient. Patients in this study relied on their own limited knowledge which often persuaded them to seek and engage with a variety of health communication strategies. The need for guidance arises at various stages of the patient’s trajectory and adaptation to a chronic illness and such needs are influenced by perceived self-efficacy related to the chronic illness (Inzucchi et al. 2012).

The art of communicating health information integrates various contextually appropriate concepts that support decoding and comprehension of messages conveyed (Harvey & O’Brien 2011). The frankness of some health care workers’ personality, although perceived as threatening, resulted in positive outcomes. Although this may not be the case for most patients, health care workers may need to calibrate their own personality in relation to the patient at hand for the patient’s maximal benefit (Stanyon et al. 2016).

The complexity of communicating health information infiltrates through some teaching aids used throughout the health delivery system in the Free State. Printed media is a common method of conveying health information and has received phenomenal attention in countries with high functional literacy (Leonard et al. 2018). However, printed media in low- and middle-income countries has presented several challenges related to its acceptance and application, some of which were confirmed by the participants of this study. Common to low- and middle-income countries is a higher readability level of printed media when compared with the literacy level of its intended participants. Readability of the printed media is associated with the level of comprehension, influenced by the language used to communicate health information (Krige & Reid 2017). The South African context compounds this non-alignment between the readability level of printed media, the heterogeneity of the population and the literacy level of patients, as patients with diabetes are usually older, underprivileged and of poor education levels.

Peer support and education, although unstructured and informal, seemed to be a common technique used in transmitting health information among patients with diabetes within various communities in the Free State. Likewise, in the field of HIV management, the concept of ‘expert patients’ has supported health care workers in delivering health information related to adherence and self-care for other HIV-positive patients (Landes et al. 2017). Such approaches may need to be devised and strengthened in areas of high disease burdens like South Africa.

Patients with diabetes are expected to adapt their lifestyle based on the health information they have received for improved health outcomes. The adaptation of the patients and transformation of their lifestyle were influenced by several factors that may have been extrinsic or intrinsic. Patients need health information that is tailor-made to their own individual and specific needs (Dublon et al. 2017). Such tailor-made information may come from exploring patient challenges and prescribing exactly what they need. External and internal factors may influence the adaptation of health information negatively, resulting in poor health outcomes.

The study revealed that the patients were adapting their lifestyle based on the health information they received. This modification was perceived in their nutrition and diet and in some outcomes of their lifestyle. The patients diagnosed with diabetes rely on the social environment to self-manage; however, critical to such environment is the ability to generate positive thoughts and acceptance, thus improving their general mental ability to achieve positive health outcomes (Cornett 2009).

## Conclusion

Diabetes mellitus is a life-threatening lifestyle disorder and patients are central to the management of their disease. This study is part of the primary studies of an overarching study engaged in the development of a complex health dialogue model for patients with diabetes in the Free State. Through a qualitative descriptive design, we described the perceptions of patients diagnosed with diabetes mellitus regarding the health communication strategies in the Free State.

The design, development and implementation of the health dialogue model for patients with diabetes in the Free State should be premised on the notion that patients value the interactions with their health care providers and other patients with diabetes, be aware that opportunities to ask questions related to their health are not always presented. Information sharing among patients is essential in self-management and such information should be tailor-made to the specific needs of the patients which may include their health and language needs.
